# 587. Research and Development of Image Recognition AI to Estimate Bacterial Species using Gram Stain Findings in Aerobic and Anaerobic Blood Culture Bottle

**DOI:** 10.1093/ofid/ofad500.656

**Published:** 2023-11-27

**Authors:** Isao Miyatsuka, Kei Yamamoto, Goh Ohji, Norio Ohmagari, Masami Kurokawa, Kei Furui Ebisawa, Kenichiro Ohnuma, Mari Kusuki, Mitsutaka Nakada, Shogo Maeta

**Affiliations:** CarbGeM Inc., Shibuya-ku, Tokyo, Japan; National Center for Global Health and Medicine, Shinjuku-ku, Tokyo, Japan; Kobe University Graduate School of Medicine, Kobe, Hyogo, Japan; National Centre for Global Health and Medicine, Shinjuku, Tokyo, Japan; National Center for Global Health and Medicine, Shinjuku-ku, Tokyo, Japan; Kobe University Hospital, Kobe, Hyogo, Japan; Kobe University Hospital, Kobe, Hyogo, Japan; Kobe University Hospital, Kobe, Hyogo, Japan; CarbGeM Inc., Shibuya-ku, Tokyo, Japan; CarbGeM Inc., Shibuya-ku, Tokyo, Japan

## Abstract

**Background:**

Background

In the highly fatal infectious disease of bacteremia, the ability to select antimicrobial agents at an earlier time is critical not only for early patient recovery, but also for reducing the development of resistant organisms. Gram staining of blood cultures is a useful test for early selection of antimicrobial agents, but it requires some skill in deciphering. Therefore, we developed an image analysis system for Gram stain images of blood cultures and have verified whether it is possible to estimate the bacterial species to facilitate the initial selection of antimicrobial agents, regardless of the level of proficiency.

**Methods:**

Method

Slides and bacterial species-identification information from an anonymized Gram stain registry at two medical institutions, National Center for Global health and Medicine (NCGM) and Kobe University Hospital (KUH), were used for the study. 1113 cases of aerobic bottles and 1060 cases of anaerobic bottles were included. Mock-specimens were used for the rare bacteria. A total of 23,947 images were generated by capturing the observation field of view of an optical microscope with a smartphone. Table 1 shows the bacterial classification of aerobic bottles and Table 2 shows the bacterial classification of anaerobic bottles. The data were divided into training data and test data at a ratio of 8 to 2 for each category.

**Table 1.**
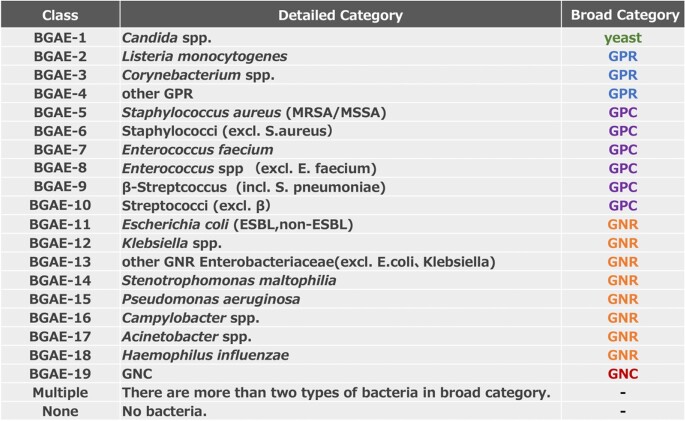
A total of 16 categories of bacteria were used for classification of aerobic bottles

**Table 2.**
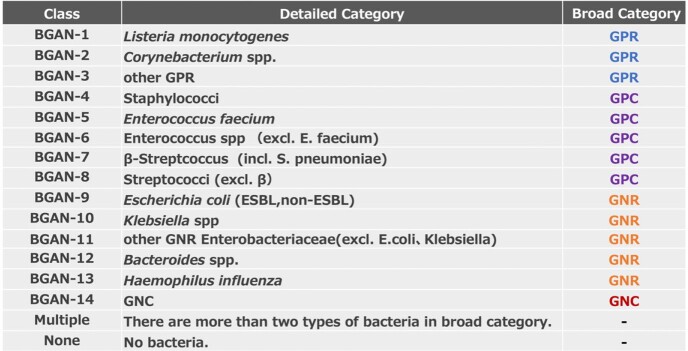
A total of 16 categories of bacteria were used for classification of anaerobic bottles

**Results:**

The macro-average recall (sensitivity) and accuracy of the test data were 64% and 82% for aerobic bottles and 68% and 86% for anaerobic bottles, respectively. The results showed the possibility of classifying the performing bacterial species with an accuracy of over 70% using only Gram stained images via image recognition AI. For aerobic bottles in particular, the seven categories of GNR, GNC, GPR, GPC, yeast, No organism, and multiple bacteria are predicted to have 97% accuracy, and 90% macro average recall.

**Conclusion:**

The research has shown that it is possible to classify bacterial species to a certain extent only by observing the Gram stain images. Our goal is to narrow down and focus more on the clinically-meaningful bacterial species, and to improve the accuracy of our classification. Also, we are planning to make comparisons with specialists in the future.

**Disclosures:**

**Kei Yamamoto, MD**, Canon medical systems: Grant/Research Support|CarbGeM: Grant/Research Support|CarbGeM: 7090302|Fujirebio: Grant/Research Support|Sanyo Chemical Industries: Grant/Research Support|VisGene: Grant/Research Support **Goh Ohji, MD, PhD, DTMH**, CarbGeM: Advisor/Consultant

